# Factors influencing severity of recurrent malaria in a conflict-affected state of South Sudan: an unmatched case-control study

**DOI:** 10.1186/s13031-022-00463-z

**Published:** 2022-06-11

**Authors:** Israel Oluwaseyidayo Idris, Gabriel Omoniyi Ayeni, Ihoghosa Osamuyi Iyamu, Ayomide Busayo Sina-Odunsi, Yusuff Adebayo Adebisi, Justin Geno Obwoya

**Affiliations:** 1 Department of Field Operation and Project Coordination, Health Pooled Fund, Juba, South Sudan; 2 Department of Social and Preventive Medicine, V.N Karazin Kharkiv National University, Kharkiv, Ukraine; 3 Department of Population Health, Faculty of Epidemiology and Population Health, School of Hygiene and Tropical Medicine, London, UK; 4 School of Population and Public Health (SPPH), University of British Columbia, Vancouver, Canada; 5 Institute of Applied Health Sciences, University of Aberdeen, Aberdeen, UK; 6 Regional Office for the East and Horn of Africa, International Organization for Migration, United Nations Migration Agency, Nairobi, Kenya; 7 Faculty of Pharmacy, University of Ibadan, Ibadan, Nigeria

**Keywords:** Severe malaria, Primary healthcare centre, Anti-malaria treatment, Humanitarian crises, South Sudan

## Abstract

**Background:**

The burden of malaria remains the highest in sub-Saharan Africa and South Sudan is not an exception. The country has borne the brunt of years of chronic warfare and remains endemic of malaria, with increasing mortality and morbidity. Limited data still exists on factors influencing the recurrence of severe malaria, especially in emergency contexts such as South Sudan, affected by various conflicts and humanitarian situations. This study therefore aimed to investigate factors influencing severity of occurrence malaria in selected primary healthcare centres in South Sudan. This would assist and guide in malaria prevention, treatment, and eradication efforts.

**Methods:**

We conducted an unmatched case-control study using routinely collected clinic data for individuals aged 1 year and above who received a diagnosis of severe malaria at 3 primary healthcare centres (PHCC); Malual Bab PHCC, Matangai PHCC and Malek PHCC between September 15, 2019 to December 15, 2019 in South Sudan. Patient characteristics were analyzed using simple descriptive statistics. Inferential statistics were also conducted to identify the associated factors influencing recurrence of severe malaria. All analyses were conducted using R Version 3.6.2.

**Results:**

A total of 289 recurrent malaria cases were included in this study. More than half of the participants were female. Overall, the prevalence of severe recurrent malaria was 66.1% (191) while 74.4% (215) did not complete malaria treatment. Among those who did not complete malaria treatment, 76.7% (165) had severe recurrent malaria, while among those who completed malaria treatment 35.1% (26) had severe recurrent malaria (*p* < 0.001). There is a significant association between marital status (OR 0.33, 95% CI 0.19–0.56, *p* < 0.001), employment status (OR 0.35, 95% CI 0.14–0.87, *p* = 0.024), the use of preventive measures (OR 3.82, 95% CI 1.81–8.43, *p* < 0.001) and nutrition status (OR 0.22, 95% CI 0.13–0.37, *p* < 0.001). When adjusted for employment, marital status, nutritional and prevention measures in turns using Mantel–Haenszel test of association, this effect remained statistically significant.

**Conclusions:**

Our study showed that there is a high prevalence of severe recurrent malaria in South Sudan and that a significant relationship exists between severe recurrent malaria and antimalarial treatment dosage completion influenced by certain personal and social factors such as marital status, employment status, the use of preventive measures and nutrition status. Findings from our study would be useful for effective response to control and prevent malaria in endemic areas of South Sudan.

## Introduction

Communicable diseases continue to pose a significant public health threat in Africa and South Sudan is not an exception [1]. According to the findings of a systematic review on the impact of conflict on health outcomes in sub-Saharan Africa, South Sudan populations have been significantly deprived of the basic healthcare services as a result of armed conflicts. The negative effects include destruction of health facilities, killing of health workers, malnutrition with significant impact on children, increased cases of mental and psychosocial disorders, outbreaks and other public health emergencies [2]. Despite the interventions of humanitarian organizations, protracted conflict is significantly depleting and has burnt out the skilled health workforce capacity to deliver health services with only an estimated 25–30% of the South Sudanese population having access to healthcare in 2015 [3]. The effects of these years of conflicts and insecurity have distorted malaria prevention and control activities, and has placed malaria among the top five deadliest diseases and causes more than one-third of all deaths in South Sudan [4]. In fact, a recent report also revealed that 800,000 South sudanese will continue to lose access to healthcare and this has reversed efforts in malaria response in the country [5].

The World Health Organization (WHO)-World Malaria report stated that there has been a substantial achievement in the reduction of the global burden of malaria since 2010 [6]. However, a critical analysis of the reported data in recent years strongly indicates a stall in the progress between 2015 and 2019 [6]. Globally, in 2017, 219 million malaria cases were reported, compared with 214 million and 239 million cases reported in 2015 and in 2010 respectively. The falloff in the malaria burden reduction progress between 2015 and 2019 is largely attributed to the weighty malaria burden of eighteen countries inclusive of South Sudan. For example, Ghana and Nigeria, one of the eight highest malaria burden countries reported the highest absolute increases in cases of malaria in 2018 compared to 2017, while other highest burden countries reported a similar burden estimate from 2016 to 2018 except for Uganda and India. The exact burden of malaria in these countries still remains partially elusive, not excluding South Sudan [7, 8]. This is because most of the malaria-related deaths turn out majorly in the community, and not in the government owned healthcare facilities [9], where death registration is usually processed in the civil registration and vital statistics (CRVS) system [10]. In the past decade, there was an abated population at risk of malaria infection in the five regions of WHO [6, 8, 11–13]; and few countries with previous high malaria estimates are now on track towards malaria elimination reporting a slump in trend for malaria cases. China, known to have in the past, reported about30 million malaria cases in a year, now reports zero cases since 2017 [14].

The reduction in the global malaria burden was due to a scale-up of combinations of control strategies which comprise of: insecticide treated nets (ITNs) or long-lasting insecticide-treated nets (LLINs), intermittent preventive therapy for pregnant women for prevention, and indoor residual spraying, better diagnostics for case ascertainment, and effective treatments using artemisinin-based combination therapies (ACTs) [15, 16]. ITNs was not generally singled out by the majority of directly observed studies as the sole or major driver of the decline as suggested recently [17]. The proportion of children under 5 years of age sleeping under ITNs in sub-Saharan Africa, has increased to an estimated 68% (95% CI 61–72%) in 2015 from < 2% in the year 2000, although the estimates vary widely between countries [13]. Despite these gains, recent studies highlighted the fact that the distribution, ownership, and actual use of ITNs is still inefficient and potentially undermines its effectiveness [17]. Investments in health systems and improved availability of ACTs and rapid diagnostic tests (RDTs) have also played a role in the gain [18], including in South Sudan. A study in South Sudan however identified compliance to effective malaria prevention and treatment measures as a major challenge (19). Other approaches are under investigation, and some have shown promise, but have not yet been widely deployed, these include seasonal malaria chemoprevention (SMC) [19] and mass drug administration (MDA) [20]. At the time this research was conducted, no published and unpublished literature has stated the wide scale implementation of the two latter approaches in South Sudan.

It is of huge concern that despite the progress being made in the global fight against malaria, a significant proportion of the population living in malaria endemic areas often experience more than one malaria episode within a single season. The distribution and determinants of the recurrent malaria attacks have been reported to depend on the local epidemiological environment. For example, in hyper-endemic areas of Africa, children suffer repeated malaria episodes every 4–6 weeks [21–23]. In low malaria transmission areas of Africa, it was posited that a person is likely to experience 1–3 episodes of malaria in a year [24]. Also, a severe malaria study finding showed that mortality increased with repeated convulsion [25]. With severe recurrent malaria, the chance of repeated convulsion is higher and thus increases in mortality.

It is important to note that several studies have been conducted on risk factors of malaria and/or severe malaria, however, most of the previous studies examined the risk factors of malaria or severe malaria with little or no attention given to the factors of severity of recurrent severe malaria. Some studies examined malaria and severe malaria in relation to use of malaria preventive and control measures [26–30]. One of the studies examined the social determinants of malaria but it did not evaluate the relationship between social determinants and recurrent severe malaria [31]. Hence, limited data thus still exists on factors influencing the recurrence of malaria and the severity of its recurrence especially in emergency contexts such as South Sudan, affected by various conflicts and humanitarian situations, which limit the health systems and research optimization [12]. South Sudan, a fragile and conflict-affected country, is believed to be among the farthest from achieving the Millennium Development Goals (MDGs) on health. Estimations show that more than half of the world’s poorest people are residing in countries with the most fragile governing system [32–34]. It is beneficial to know and understand factors responsible for recurrence of malaria if the goal of controlling the endemic nature of malaria in South Sudan is to be achieved., This will contribute to the Global Malaria Programme GMP efforts to control and eliminate malaria.

This study aimed at identifying predisposing factors associated with severe recurrent malaria in the capital town of Lakes State in South Sudan.

## Methods

### Study setting

This study was conducted in Lake’s state, a part of the Bahr el Ghazal region, central area and one of the 10 states of South Sudan. The climate of South Sudan is tropical with average temperature of 200 to 370 C, relative humidity between 26% and 88% [35]. Rainy seasons differ from regions and locations in the country with April to November being the peak of the rainy season and December to March the dry season. Upper Nile and Bahr el Gazal regions receive rains from 700 to 1300 mm, while South-Eastern tip of Eastern Equatoria receives less than 200 mm of rain. Western Equatoria and highland areas in Eastern Equatoria receive between 1200 and 2,200 mm of rain [36]. In rainy seasons, the lowlands and river bank areas get heavily flooded, acting as breeding grounds for mosquitoes [37]. These factors, among others, resulted in the increased malaria prevalence during the rainy season as a suitable season for breeding of malaria vectors.

Since 2005, the former Western Lakes state region, one of the most insecure areas of South Sudan has experienced brutal conflict which has claimed thousands of lives and driven poorly estimated people from their homes. From land, agricultural and water disputes to armed robberies, these violent occurrences have sustained the insecurity in Western Lakes state [38]. This has been worsened by re-occurrence of natural disasters e.g. heavy (destructive) flooding during the wet season [39]. The former Western Lakes State includes four counties - Rumbek centre, Wulu, Rumbek North, Rumbek East counties. Rumbek centre county is the capital and politico-economic centre of the state; it is also known as the highest populated county in the state of about 232,752 people in 2017. Rumbek centre county has only three functioning government-owned primary healthcare centres (PHCCs) and 13 primary healthcare units (PHCUs). Due to the high insecurity and looting of health facilities in the county, the government’s efforts in strengthening the county health system are still faced with many problems including lack of human resources, poor and vandalised infrastructure, lack of storage and also shortage of drugs and supplies, increased workplace injuries, increased absenteeism and shortage of staff. However, efforts are being made to address these challenges with the present use of a “contracting out” model to international non-governmental organizations (INGOs) to support and strengthen the county health department in provision of a stable and quality primary health services in the country.

### Study design

We conducted an unmatched case-control study of routine clinic data collected for individuals aged 1 year and above who received a diagnosis of recurrent malaria at the three primary healthcare centres (PHCCs): Malual Bab PHCC, Matangai PHCC and Malek PHCC between September 15, 2019 to December 15, 2019 in Rumbek Centre County, Lakes state, South Sudan as those are the only three functioning government-owned PHCCs in the county. The South Sudan’s essential package of health services (ephs) guidelines do not support treatment of severe malaria cases in PHCUs and so, PHCUs were not included as a study site.Survivor sampling technique was adopted in the recruitment of controls and cases; cases and controls are persons from the same population who visited the aforementioned health facilities for curative consultation during the study period.

### Definition of cases and controls

The study population consisted of all cases of malaria diagnosed as a recurrent malaria case using the antigen Plasmodium lactate dehydrogenase-rapid diagnostic test (RDT) for malaria. Date of previous consultation visitation by the patients were reported by the patients or their carer and were all verified on the out-patient department (OPD) registers in the PHCCs. Recurrent malaria case was defined as a subsequent malaria case that occurred after 7 days to 43 days of the first dose of the anti-malaria treatment with all differential diagnosis eliminated by respective laboratory investigations. This definition was adopted based on a recent posit that the clinical efficacy of clearing falciparum parasites can be observed within 5 days of artemisinin-based combination therapy (ACT) [40]. Controls were also cases of malaria diagnosed as recurrent malaria cases using the antigen Plasmodium lactate dehydrogenase-rapid diagnostic test (RDT) with no clinical history of severe malaria. All patients were only categorized into under 5 and 5 years old or above to reduce the occurrence of self-reported age bias with a cut-off age of 1 year old. Arguably, infants are reported to be less susceptible to clinical malaria, due to the transferred immunity from their mother during exclusive breastfeeding [41], and we hypothesize that this could be associated with missed diagnosis of previous asymptomatic malaria episodes in these infants. In our study context, children under 1 year old especially infants below 3 months old were difficult to be timely diagnosed for severe malaria and are often delayed diagnosis because clinical presentation mostly mimics other diseases, such as sepsis. This is in addition to the frequent false-negative result of the RDT commonly used to diagnose malaria among these age groups. A prior study suggests that these factors may have led to a high home deaths of malaria in this age-group [42]. We adopted the above recurrent malaria definition into the South Sudan’s guideline for definition of severe malaria case to define our a severe recurrent malaria case as an individual who have revisited the PHCC having fever (> 38 °C) and vomiting (> 1-day duration) with any of the following symptoms; convulsions, abnormal sleep, inability to drink, coma, severe anaemia, jaundice, coloured urine, bleeding, dyspnoea and shock; and have tested positive with RDT after 7 days to 43 days of the first dose intake of the prescribed anti-malaria treatment [43].

### Data collection procedure

Past medical history from eligible participants (cases and controls) were identified through records at the PHCC. Data on sociodemographic, socioeconomic, and behavioural characteristics of the case and control patients were also collected directly from the patient by the research team using a structured closed-end questionnaire in a face-to-face interview. Lastly, all the primary quantitative data was entered into Microsoft Excel and later imported into R Version 3.6.2 for analysis.

### Data analysis

Bivariate analysis was conducted to determine associations between the main exposure variable (incomplete malaria treatment) and other independent variables with the primary outcome (severe recurrent malaria), using Chi-square test (of Fisher’s exact test in cases of small subgroup sample sizes). Crude odds ratios (OR) with 95% confidence intervals (CI) were estimated using binary logistic regression to quantify the association between severe recurrent malaria and other independent variables including the main exposure variable. The Mantel-Haenszel method was used to test the association between incomplete malaria treatment and severe recurrent malaria, stratifying for each of these potential confounding variables in turn. Crude odds ratio obtained from association between main exposure and primary outcome was compared with Mantel-Haenszel odds ratio for confounders’ identification. Homogeneity test was carried out to compare stratum-specific odds ratios to identify effect modifiers.

Multivariable logistic regression models were used to estimate the causal effect of incomplete malaria treatment on severe recurrent malaria while controlling for potential confounding and calculated 95% confidence intervals for these estimates. We considered *p* values less than 0.05 as statistically significant and all analyses were conducted using R Version 3.6.2.

## Results

A total of 289 participants were included in this study. As summarized in Tables 1 and 64.2% (185) of the participants were females, 38.1% (110) were under 5 years of age, 26.0% (75) were married, 47.8% (138) ate once or twice daily and 88.9% (257) had environmental measures and/or insecticide treated nets as malaria prevention measures. Overall, the prevalence of severe recurrent malaria was 66.1% (191) while 74.4% (215) did not complete their anti-malaria treatment. Among those who did not complete anti-malaria treatment, 76.7% (165) had severe recurrent malaria, while among those who completed anti-malaria treatment, 35.1% (26) had severe recurrent malaria (p < 0.001).

**Table 1:** Distribution of study sample characteristics and the risk of severe malaria

Variable	Severe recurrent malaria		Crude Odds Ratio	*p* value
	No (%)	Yes (%)	(95% CI)	
Malaria treatment				
Complete treatment	48 (49.0)	26 (13.6)	Ref	
Incomplete treatment	50 (51.0)	165 (86.4)	6.09 (3.47, 10.93)	< 0.001*
Age				
Less than 5 years	32 (32.7)	78 (40.8)	Ref	
5 years and above	66 (67.3)	113 (59.2)	0.7 (0.42, 1.17)	0.176
Sex				
Female	58 (59.8)	127 (66.5)	Ref	
Male	39 (40.2)	64 (33.5)	0.75 (0.45, 1.25)	0.263
Marital status				
Single	58 (59.2)	156 (81.7)	Ref	
Married	40 (40.8)	35 (18.3)	0.33 (0.19, 0.56)	< 0.001*
Employment status				
Unemployed	86 (87.8)	182 (95.3)	Ref	
Employed	12 (12.2)	9 (4.7)	0.35 (0.14, 0.87)	0.024*
Rapid diagnostic test				
Negative	13 (13.3)	40 (20.9)	Ref	
Positive	85 (86.7)	151 (79.1)	0.58 (0.28, 1.11)	0.113
Feeding habit				
Once/twice daily	24 (24.5)	114 (59.7)	Ref	
At least thrice daily	74 (75.5)	77 (40.3)	0.22 (0.13, 0.37)	< 0.001*
Preventive measures				
None	20 (20.4)	12 (6.3)	Ref	
Environmental sanitation and/or ITN	78 (79.6)	179 (93.7)	3.82 (1.81, 8.43)	< 0.001*
N = 289				

*Significant: *p* < 0.05

Among those who were married, 46.7% (35) had severe recurrent malaria compared with 81.7% (156) of those who were single. Married participants were less likely to have severe recurrent malaria compared with those who were single (OR 0.33, 95% CI 0.19–0.56, *p* < 0.001). Similarly, 42.9% (9) of employed participants had severe recurrent malaria compared with 67.9% (182) of unemployed participants. Employed participants were therefore less likely to have recurrent malaria compared with those who were unemployed (OR 0.35, 95% CI 0.14–0.87, *p* = 0.024). Furthermore, 51.0% (77) of participants who ate at least twice daily had severe recurrent malaria compared with 82.6% (114) of those who ate a maximum of one meal daily. This association was significant as those who ate at least twice daily were 0.2 times as likely to have severe malaria compared to those who ate a maximum of one meal daily (OR 0.22, 95% CI 0.13–0.37, *p* < 0.001). Conversely, 69.6% (179) of participants who had at least one malaria preventive measure developed severe recurrent malaria compared with 37.5% (12) of those who had no prevention. Therefore, those with at least one preventive measure were 3.8 times more likely to have severe recurrent malaria compared with those who had no prevention measure (OR 3.82, 95% CI 1.81–8.43, *p* < 0.001).

In the bivariate analysis, there was a strong statistical association between incomplete malaria treatment and the occurrence of severe recurrent malaria (Table 2). Participants with incomplete malaria treatment were more likely to have severe recurrent malaria compared with those who completed malaria treatment (OR 6.09, 95% CI 3.47–10.93, *p* < 0.001). When adjusted for employment, marital status, feeding habit and prevention measures in turns using Mantel-Haenszel test of association, this effect remained statistically significant at (aOR 5.73, 95% CI 3.22–10.22, *p* < 0.001), (aOR 6.44, 95% CI 3.54–11.75, *p* < 0.001), (aOR 9.03, 95% CI 4.52–18.00, *p* < 0.001) and (aOR 5.52, 95% CI 3.07–9.90, *p* < 0.001) respectively. In multivariable analysis (Table 3), adjusted for employment, marital status, nutritional and preventive measures, those with incomplete malaria treatment were more than 10 times as likely to have severe recurrent malaria compared with those with complete malaria treatment (aOR 10.62, 95% CI 4.95–24.54, *p* < 0.001).

**Table 2:** Adjusted estimates of the odds ratio for the association between incomplete malaria treatment and severe recurrent malaria estimated using the Mantel–Haenszel method (n = 289)

Variable	Odds ratio (95% CI)	*p* value
Crude Association	6.09 (3.47, 10.93)	< 0.001*
Association Adjusted For		
Employment status	5.73 (3.22, 10.22)	< 0.001*
Marital status	6.44 (3.54, 11.75)	< 0.001*
Feeding habit status	9.03 (4.52, 18.00)	< 0.001*
Preventive measures	5.52 (3.07, 9.90)	< 0.001*

*Significant: *p* < 0.05

**Table 3:** Adjusted estimate of the odds ratio for the association between incomplete malaria treatment and Severe recurrent malaria using multivariable Logistic regression models (n = 289)

Model	Variables	Odds Ratio (95% CI)	*p* value
1	Malaria treatment	6.09 (3.47, 10.93)	< 0.001*
2	Malaria treatment + employment status	5.76 (3.25, 10.41)	< 0.001*
3	Malaria treatment + employment status + marital status	6.45 (3.56, 11.98)	< 0.001*
4	Malaria treatment + employment status + marital status + feeding habit status	12.84 (6.04, 29.64)	< 0.001*
Final	Malaria treatment + employment status + marital status + nutritional status + preventive measures	10.62 (4.95, 24.54)	< 0.001*

*Significant: *p* < 0.05

## Discussion

While there is dearth of data on severity of recurrent malaria, this study has revealed that most (66.1%) malaria recurrence cases appeared in its severe episode, wherein the study further identified the factors that influence recurrence of malaria in a severe form. Our results revealed that the prevalence of severe recurrent malaria among our study participants was higher in the five years old and above population when compared to the under-five years old population. This is consistent with previous studies; however, more sub-classifications of age were applied in those studies [26, 28, 44]. Herein, It is important to conduct further investigation to detect if the prevalence of severe recurrent malaria among adult participants was as a result of waned immunity (being an area of malaria endemicity), or a factor of drug resistance or otherwise. According to WHO, in areas of moderate or intense malaria transmission, partial immunity is developed over years of exposure especially among adults; and while it never provides complete protection, it does reduce the risk that malaria infection will cause severe diseases [45, 46]. In this view, further investigation on this may be suggested, in line with the WHO request for continuous monitoring of drug efficacy [45, 46], as the possibility of gradual emergence of drug resistant malaria infection may not be ruled out, and this may undermine the malaria control efforts.

In addition to the above, our study findings also revealed that 76.7% of those who did not complete their treatment had severe recurrent malaria, unlike the 35.1% seen among those who completed their treatment. A recent study has shown 24.1% recurrence of malaria with at least one episode within 180 days of treatment completion were attributed to treatment failure [47]. Other studies showed 0-13.5% malaria recurrence following treatment with standard regimen [48–50]. The finding of this study further revealed that ‘completion or not’ of the South Sudan standard malaria treatment regimen by patient was a significant factor to the severity of malaria recurrence among the population (see Appendix for the South-Sudan standard malaria treatment regimen). The finding showed significant association irrespective of the employment, marital, feeding habit, or preventive measure access status of the participants. Past malaria studies have shown non-completion of treatment to be a predisposing factor of severe recurrent malaria [26, 51]. As the Government of South Sudan through the Ministry of Health (MoH) and various partners are prioritizing by investing enormous resources in the prevention and control of malaria including increasing access to effective treatment, more efforts are needed in addressing the factors influencing adherence to anti-malaria treatment in these settings.We strongly recommend thatsubsequent studies should aim to identify the factorsassociated with adherence per each type of theMoH recommended anti-malaria treatment regimen in protracted conflict settings like the Lakes state of South Sudan. In fact, it is a joint-call to clinicians and other major health stakeholders to review and recommend strategies and best-practices for patients to cope with their medications’ completion accordingly as prescribed, on time, and also for healthcare providers to follow-up/track completion of prescribed medication or dosage prescribed to patients respectively. Also, government and major partners should increase focus on monitoring of antimicrobial resistance (AMR) across regions in relation to severe malaria, so as to be able to timely implement actions in the situation of any negative trend of being identified. Furthermore, while treatment guidelines recommend followup (including on prescribed drug completion) as part of interventions in patients’ care, there is the need for its inclusion among care indicators to be monitored and reported by health managers monthly/quarterly by health managers. However, considering the limitations of insecurity, limited resources and infrastructures in the country, the monitoring focus could commence with moderate-severe conditions managed at primary health care centres.

The finding of this study showed that a good proportion of the population had access to insecticide treated bednets and other environmental preventive measures. This suggested the population in the study area might have benefitted from the investment made towards improving access to proven preventive measures for malaria, especially the treated mosquito nets. Previous studies have shown that access to an effective utilization of treated mosquito nets significantly prevents the occurrence of both uncomplicated malaria [13, 27, 28], and complicated malaria [26]. The findings of this study showed that participants who had at least one malaria preventive measure developed severe malaria compared with those who had no prevention. Those with at least one preventive measure were 3.8 times as likely to have severe recurrent malaria compared with those who had no prevention measure. It could become necessary for government and major partners to monitor and respond to how effective any of the preventive measures against malaria/severe malaria is among the benefiting population, as there may be a variance of poor compliance to effective utilization of these preventive measures among the different socio-economic groups and contexts. It will be useful to further investigate the type and quantity of preventive measures the population has accessed and utilised. Furthermore, the chance of false prevention might have existed among the population, such as using defective preventive measure items (e.g. insecticide treated bednets with minor tears), hence increasing their exposure to having recurrent severe malaria. The findings from previous studies showed that attitude and inadequate knowledge of the effective use of malaria preventive and control measures affect compliance and the outcome [26–30]. Among available preventive measures, WHO recommends protection for all people at risk of malaria with effective malaria vector control – insecticide-treated mosquito nets and indoor residual spraying which are effective in a wide range of circumstances [45].

Furthermore, the outcome of this study was found to be dependent on two socio-demographic factors- marital and employment statuses. In contrast to a published study finding that reported a significant relationship between gender, and prevalence of malaria [31], our study indicated that there was no strong evidence for the association between gender and the occurrence of severe malaria. Further,our study has revealed that severity of recurrent malaria had a significant relationship with marital status. Married participants were less likely to experience a severe episode of recurrent malaria when compared with those who were unmarried. This is consistent with a published study conducted among head porters in Agbogbloshie market in the Greater Accra region of Ghana. This finding can be supported with the postulation that married persons could lend more care and protection to one another at home including preventive health care such as providing reminders on medication, providing support in the proper utilization of treated mosquito nets among others, unlike unmarried persons. Most of the previous studies that examined the social determinants of malaria did not evaluate the relationship between severe (recurrent) malaria and marital status [52, 53]. The finding on marital influence on the severity of malaria recurrence could be utilized in health education and promotion to reinforce a sense of health responsibility and positive health attitudes in homes.

Our study also revealed that the severity of malaria recurrence was found to be more likely among those who are not employed when compared with those gainfully employed. The possibility of a reduced positive emotional state of mind caused by unavailability or limited financial resources could have affected the health seeking behavior of the unemployed population. Furthermore, the finding of the study showed significant association between participants who ate at most once or twice daily had severe recurrent malaria compared with those who ate a minimum of thrice meal daily. This can be argued that participants with access to more or adequate meals should have more relevant nutrients and better health status to prevent and /or respond to illness. This did not contradict the fact that there could be a possibility that those who had access to more meals might not have had access to a balanced or quality diet, hence limited benefit to their body health systems; and therefore could have reduced the protective effect against the severity of recurrent malaria in our study (adj OR 9.03, 95% CI 4.52-18.00, *p* < 0.001). This study should have but could not further investigate the type and quality of meal the participants had; hence, it will be beneficial to the government of malaria endemic nations if further studies can explore the type and effect of meal quality on the recurrence of severe malaria. The findings of various studies conducted suggested poor feeding as a result of poverty to be a factor influencing malaria occurrence [52–54]. Available evidence alluded to the fact that where malaria thrives most, human societies have prospered least, and this suggests that malaria and poverty are closely linked [55, 56]. Programmes which focus on sustainable empowerment of families and communities economically may contribute to reducing the incidence of recurrent severe malaria and should be more advocated in the area and other areas with similar epidemiological patterns for severe malaria. Furthermore, beyond integrating nutrition programmes with severe malaria care and other PHC programmes as presently being seen [57], extending or linking it further with livelihood programmes could further help and facilitate the goal of controlling and eliminating malaria in the country.

To conclude, this study has further revealed the multi-faceted nature of factors influencing severity of re-occurrence of malaria and this will require coordinated multi-approach interventions by government and all major stakeholders to timely achieve the goal of ending malaria. Recognizing that a lot of investment has been made on malaria programmes in the country and more still ongoing, prioritizing monitoring for efficacy/effectiveness and compliance to both antimalarial treatment and malaria preventive measures in the country and similar contexts is highly essential to derive and/or guarantee value for investments made.

## Study limitation

The weakness of our study include that the use of patient-provided clinical data may have exposed the study to recall bias. However, considering that data was collected without specific attention drawn to our research question, we believe this bias to be minimal. Further, our retrospective use of the data implied that the spectrum of covariates that could be controlled in this study were limited. Although, as this study is one of the first of its kind, we find it inevitable to admit that as part of the weaknesses of our study was the lack of validity test of the questionnaire prior to its usage. Questions on the specific initially used treatment and hospitalization status before the compliance variable were omitted in the questionnaire as we assumed that all treatment prescribed and received would have been in accordance with the South Sudan treatment guideline (see supplementary file). In that respect, this study can be seen as a call to the WHO advisory committee in designing a uniform questionnaire in regard to this topic. It would have been of additional impact if our study was capacitated to detect and reclassify the recurrent malaria cases into: relapse malaria case, recrudescence malaria cases, reinfection malaria cases. Although, we believe that the observed severe recurrent malaria cases could be more of a reinfection or relapse case than a recrudescence case. Lastly, we acknowledge that the use of antigen Plasmodium lactate dehydrogenase- RDT instead of the gold-standard microscopy blood film malaria test may have resulted in a number of false positive recurrent malaria cases in the study. Past study results have shown that these RDTs can detect antigen Plasmodium lactate dehydrogenase in the blood plasma as positive 28 (18–35) days after an antimalarial treatment (with an overall sensitivity of 87.6% (85.9–89.2%), and specificity of 75.8% (74.4–77.1%)). Although, this depends on the age and type of anti-malaria treatment received [58, 59].

## Conclusions

This study indicated a high prevalence of severe recurrent malaria in South Sudan, with a significant relationship to certain characteristics of individuals within the population such as marital status, employment status, the use of preventive measures and feeding habits. There was significant association between the severity of malaria recurrence and non-completion of prescribed malaria treatment regimen. This study data will no doubt be useful for the prevention and control strategy of malaria in endemic areas of South Sudan. Further studies on factors influencing anti-malaria treatment adherence, prevalence of anti-malaria drug resistance to malaria and compliance to preventive measures is recommended.

## Data Availability

On reasonable request from the corresponding author, datasets used in this study are readily available.
